# Rescue of Infectious Rotavirus Reassortants by a Reverse Genetics System Is Restricted by the Receptor-Binding Region of VP4

**DOI:** 10.3390/v13030363

**Published:** 2021-02-25

**Authors:** Alexander Falkenhagen, Marno Huyzers, Alberdina A. van Dijk, Reimar Johne

**Affiliations:** 1 Department of Biological Safety, German Federal Institute for Risk Assessment, 10589 Berlin, Germany; reimar.johne@bfr.bund.de; 2 Human Metabolomics, Faculty of Natural and Agricultural Sciences, North-West University, 2531 Potchefstroom, South Africa; gerno.huyzers@gmail.com (M.H.); albie.vandijk@nwu.ac.za (A.A.v.D.)

**Keywords:** rotavirus, reassortment, VP4, chimeric, plasmid-based reverse genetics system

## Abstract

The rotavirus species A (RVA) capsid contains the spike protein VP4, which interacts with VP6 and VP7 and is involved in cellular receptor binding. The capsid encloses the genome consisting of eleven dsRNA segments. Reassortment events can result in novel strains with changed properties. Using a plasmid-based reverse genetics system based on simian RVA strain SA11, we previously showed that the rescue of viable reassortants containing a heterologous VP4-encoding genome segment was strain-dependent. In order to unravel the reasons for the reassortment restrictions, we designed here a series of plasmids encoding chimeric VP4s. Exchange of the VP4 domains interacting with VP6 and VP7 was not sufficient for rescue of viable viruses. In contrast, the exchange of fragments encoding the receptor-binding region of VP4 resulted in virus rescue. All parent strains and the rescued reassortants replicated efficiently in MA-104 cells used for virus propagation. In contrast, replication in BSR T7/5 cells used for plasmid transfection was only efficient for the SA11 strain, whereas the rescued reassortants replicated slowly, and the parent strains failing to produce reassortants did not replicate. While future research in this area is necessary, replication in BSR T7/5 cells may be one factor that affects the rescue of RVAs.

## 1. Introduction

Rotavirus of species A (RVA), a member of the family *Reoviridae*, is a non-enveloped virus with eleven dsRNA genome segments. Infections with RVA are the most common cause of severe gastroenteritis in young children worldwide and resulted in an estimated 128,500 deaths among children younger than 5 years in 2016 [1]. RVA infections are also widespread in wild animals and livestock, in which they can cause similar symptoms as in humans [2,3,4]. Interspecies transmission and reassortment events favor the emergence of novel RVA strains with new phenotypic properties, such as altered replication potential, immunogenic profile, or host range [5,6,7].

Especially the viral outer capsid protein VP4, which mediates viral entry and is a major antigenic determinant, plays an important role in host cell tropism [8]. VP4 forms trimeric spikes that are anchored in a cavity formed by VP6 and VP7. The VP4 spikes are comprised of an N-terminal globular head region, a central body and stalk region, as well as a C-terminal foot region [9]. The first N-terminal amino acid residues and the foot region are located in the cavity formed by VP6 and VP7, while the head region as well as the body and stalk region protrude from the outer layer of the virus capsid [9]. To acquire efficient infectivity, VP4 must undergo tryptic cleavage, which generates an N-terminal receptor-binding fragment referred to as VP8* and a C-terminal fragment called VP5* [10]. A binary typing system based on the nucleotide sequences of genome segments encoding the VP7 glycoprotein (G genotypes) and the protease-sensitive VP4 (P genotypes) is commonly used [11]. Some animal RVAs, such as simian rhesus rotavirus, simian SA11, chicken Ch-1, and turkey Ty-1 were shown to require the presence of sialic acid on the cell surface for efficient infection [12]. However, the majority of RVAs can infect cells independent of sialic acid. The binding of human RVA VP8* to different glycans has been demonstrated in vitro [13,14,15]. Despite ongoing efforts, the receptor requirements for human RVAs in cell culture are still not completely understood [13,15,16,17,18]. However, it has previously been shown that RVAs can utilize different receptors depending on the cell type that is infected [18]. Despite the central role of VP4 in infection, little is known about the requirements for successful VP4 reassortment.

Several efforts have been made to develop genetic systems that allow a targeted generation of RVA reassortants. For some genome segments, a reverse genetics system (RGS) using helper viruses has successfully been established [19,20,21]. While each system has its own advantages and disadvantages, those systems generally require the isolation of reassortants by negative selection of the helper virus. Therefore, the establishment of an entirely plasmid-based reverse genetics system, without the need of a negative selection procedure, was a milestone for rotavirus research [22]. This RGS is based on the transfection of a T7 RNA polymerase-expressing producer cell line with eleven plasmids each encoding one genome segment under transcriptional control of the T7 promoter. In the original system, two helper plasmids enabling RNA capping and an additional plasmid leading to the fusion of transfected cells and increased virus recovery are co-transfected. Then, the transfected cells are co-cultured with a receiver cell line that is susceptible to rotavirus infection. Using this system or modifications thereof, such as the presence or absence of different helper plasmids and/or the utilization of different receiver cell lines, infectious virus of two simian strains (SA11 and RRV), three human strains (KU, Odelia, and CDC-9), several reassortants, as well as gene-modified RVAs, could be generated [22,23,24,25,26,27,28,29,30,31,32,33,34]. 

We previously described the successful rescue of reassortant viruses containing VP4 from a diverse set of RVAs in the backbone of simian SA11 using a plasmid-based reverse genetics system [35,36]. However, we were unable to rescue reassortants containing VP4 from one human and one avian cell culture-adapted strain as well as from two primary African human RVA strains [35,36]. Here, we designed a series of plasmids encoding chimeric VP4 proteins in order to determine potential reasons for the failure to rescue these reassortants. Additionally, the parent cell culture-adapted viruses and the generated reassortants were characterized according to their growth kinetics in the cell cultures used for the transfection of plasmids and for the subsequent passaging of generated viruses. The results indicate that the receptor-binding domain of VP4 is responsible for the observed restrictions and that the replication efficiency in the transfected cell line may be one factor limiting the generation of reassortants.

## 2. Materials and Methods

### 2.1. Cell Lines and Viruses

BSR T7/5 cells [25] and MA-104 cells were kindly provided by Karsten Tischer (Free University of Berlin, Germany) and by the European Collection of Authenticated Cell Cultures (Salisbury, United Kingdom), respectively. All cell culture reagents and cell culture conditions were described previously in detail [36]. RVA/Chicken-tc/GER/02V0002G3/2002/G19P[30], RVA/Turkey-tc/GER/03V0002E10/2003/G22P[35], and RVA/Human-wt/USA/Wa/1974/G1P[8] were kindly provided by Peter Otto (Friedrich-Loeffler-Institute, Jena, Germany) and are referred to as chicken 2G3, turkey 2E10, and human Wa, respectively. RVA/Simian-tc/ZAF/SA11-L2/1958/G3P[2] (simian SA11) was generated by reverse genetics using the plasmids described below.

### 2.2. Plasmids

The plasmids encoding the eleven simian SA11 genome segments as well as the three helper plasmids pCAG-D1R, pCAG-D12L, and pCAG-FAST-p10 were a kind gift from Takeshi Kobayashi [12] and were obtained from Addgene (Watertown, MA, USA). The plasmids encoding VP4 from chicken 2G3, turkey 2E10, human Wa, RVA/Bat-wt/CMR/BatLy03/2014/G25P[43] (Bat BatLy03), RVA/Human-wt/MOZ/0060a/2012/G12P[8] (human Moz60a), and RVA/Human-wt/MOZ/ 0308/2012/G2P[4] (human Moz308) were described previously [35,36]. An overview of all unmodified VP4-encoding genome segments is provided in Table S1. Table S2 indicates the exact location of the nucleotide substitutions for each plasmid encoding chimeric VP4. Table S3 indicates the origin of the amino acid residues in chimeric VP4 proteins. The plasmids encoding chimeric VP4-Tu/Ch, VP4-Ch/Tu, VP4-Tu/Ch/Tu, VP4-Ch/Tu/Tu, VP4-Bat/Wa, VP4-Bat/60a, VP4-Bat/308, VP4-60a/Bat, VP4-60a/Bat/Bat, VP4-Bat/60a/60a, VP4-Bat/60a/Bat, and VP4-308-A were generated using cloning techniques based on compatible restriction sites. For the generation of the plasmids encoding VP4-Si/Tu/Si, VP4-Tu-A-B, VP4-60a-A-G, and VP4-Wa-A, gBlocks gene fragments were synthesized by Integrated DNA Technologies (IDT, Coralville, IA, USA), sub-cloned into pCR4-TOPO (Thermo Fisher Scientific, Waltham, MA, USA) according to the manufacturer’s instructions, sequence verified by Macrogen (Seoul, South Korea), and cloned into the respective vector by traditional cloning methods using compatible restriction sites. The integrity of the restriction sites after cloning was confirmed by digestion with the corresponding restriction enzyme or sequencing. Sequencing primers are available upon request.

### 2.3. Generation of Reassortant Virus

Reassortant virus was generated and passaged as described previously [35,36]. A flowchart of the method is provided in [35]. BSR T7/5 cells were seeded in 6-well plates (7 × 10^5^ cell per well) and incubated for 24 hours at 37 °C and 5% CO_2_. At 90% confluency, the cells were co-transfected with 11 plasmids encoding the individual rotavirus genome segments (2250 ng for the plasmids encoding NSP2 and NSP5; 750 ng for the remaining plasmids) and three helper plasmids encoding two vaccinia virus capping enzyme subunits (750 ng each) as well as a small membrane fusion protein (15 ng) using 30 µL of TransIT-LT1 transfection reagent (Mirus Bio, Madison, WI, USA). The cells were incubated with the transfection mix for 24 h at 37 °C and 5% CO_2_ before they were washed twice with PBS, and fresh media without serum was added. Forty-eight hours later, 1 × 10^5^ MA-104 cells and a pre-made trypsin solution (0.5% trypsin/0.2% EDTA, PAN-Biotech, Aidenbach, Germany; 2 µg/mL trypsin final concentration) were added. After three days at 37 °C and 5% CO_2_, the co-cultured cells were frozen and thawed once before passaging on MA-104 cells.

### 2.4. Passaging of Reassortant Virus

A pre-made trypsin solution (PAN-Biotech) was added to the entire freeze/thaw supernatants (2 mL, 100 µg/mL trypsin final concentration), and the mixture was incubated for 1 hour at 37 °C. Confluent MA-104 cells grown in 6-well plates were washed twice with PBS, and the infection mixture was added. After 1 hour at 37°C and 5% CO_2_, the mixture was removed, and the cells were washed once with unsupplemented media. Fresh media (without serum) supplemented with a pre-made trypsin solution (PAN-Biotech, 10 µg/mL final concentration) were added, and the cells were incubated for 7 days before they were passaged again following the same protocol. The use of excess trypsin for the isolation of rotaviruses has been described previously [37]. While trypsin toxicity in uninfected cells was evident, the cytopathic effect (CPE) was clearly distinguishable. If no CPE was observed at the end of the third passage, rescue was considered negative. CPE was chosen as a readout because the occurrence of a CPE is a clear sign of effective RVA replication. All VP4 mono-reassortants that we were previously able to rescue and that we used in the present study induce CPEs that are clearly distinguishable from mock-infected control cells [35]. In contrast, no signs of CPE or viral RNA were detected for reassortants that we were previously not able to rescue [35]. As the level of CPE that is induced by primary human isolates can vary, negative and positive rescue results for chimeric constructs containing sequences from human RVAs were confirmed using qRT-PCR as described below. All rescue experiments were performed twice and in duplicate.

### 2.5. qRT-PCR, RT-PCR, and Restriction Digest Analyses

Viral RNA was extracted from freeze/thaw supernatants with the NUCLISENS easyMAG system (bioMérieux, Marcy-l′Étoile, France) and digested with RNase-free DNase (Roche, Basel, Switzerland) according to the manufacturer’s instructions. Viral RNA was detected by qRT-PCR as described previously [35]. RT-PCRs were performed using the OneStep RT-PCR Kit (Qiagen, Hilden, Germany) according to the manufacturer’s instructions. The primer sequences were as follows: VP4-Chicken-F 5′-AGAGTTGGTTACACCAAACAGAT-3′; VP4-Chicken-R 5′-AGAGTT AACAACGCCACATAGG-3′; VP4-Tu-A-F 5′-AGAGTTAACAACGCCACATAGG-3′; VP4-Tu-A-R 5′-AACCACTGTCTCACCATCCC-3′; VP4-Simian-F/VP4-Wa-A-F 5′-TCCAAACTTCACAAGACCAGTG-3′; VP4-Simian-R 5′-CGGTAACTTCTTCAC CATCACG-3′; VP4-Wa-R 5′-CGGTTACTTGTTCACCGTCA-3′. PCR amplicons were cleaned up using the Monarch DNA Gel Extraction Kit (New England Biolabs, Ipswich, MA, USA) prior to digestion with the indicated restriction enzymes according to the manufacturer’s manual (New England Biolabs). 

### 2.6. Replication Kinetics

Culture supernatants containing viruses at 2 × 10^4^ 50% tissue culture infectious doses (TCID_50_) were activated in the presence of 10 µg/mL trypsin for 30 minutes at 37 °C. Confluent MA-104 or BSR T7/5 cells grown in 6-well plates were washed twice with PBS before addition of the activated virus. Following incubation for 1.5 h at 37 °C and 5% CO_2_, the culture supernatants were removed, and 2.5 mL of fresh media containing 1 µg/mL trypsin were added. Samples (500 µL) for endpoint dilution assays were taken at the indicated time points, and the same volume of fresh media containing 1 µg/mL trypsin was added.

### 2.7. Endpoint Dilution Assays

Serially diluted virus samples were activated and used to infect confluent MA-104 cells grown in a 96-well plate as described for the replication kinetics. The infected cells were monitored for signs of CPE seven days post-infection. The Spearman and Kärber algorithm [30] was used for the calculation of the TCID_50_. All virus samples were tested in triplicate.

### 2.8. Sequence Analyses and Protein Structure Visualization

Sequence alignments were performed using the Clustal W method as implemented in MegAlign Pro (DNASTAR Inc., Madison, WI, USA). The VP4 protein sequences of RVA/Simian-tc/USA/RRV/1975/G3P[3] (simian RRV, Genbank: AAK52093.1) and human Indian G2P[4] (PDB: 5VX4) were included where indicated. Protein structures were visualized using Protean 3D (DNASTAR Inc.) on the basis of the published atomic model of an infectious rotavirus particle (PDB 4V7Q) [9].

### 2.9. Statistics

The data are presented as mean ± standard deviation (SD). To determine statistical significance, a two-tailed unpaired t test was used. Results with a *p*-value below 0.05, 0.01, or 0.001 were considered statistically significant and marked with one, two, or three asterisks, respectively.

## 3. Results

### 3.1. Chimeric Simian/Turkey VP4

**Figure 1:**
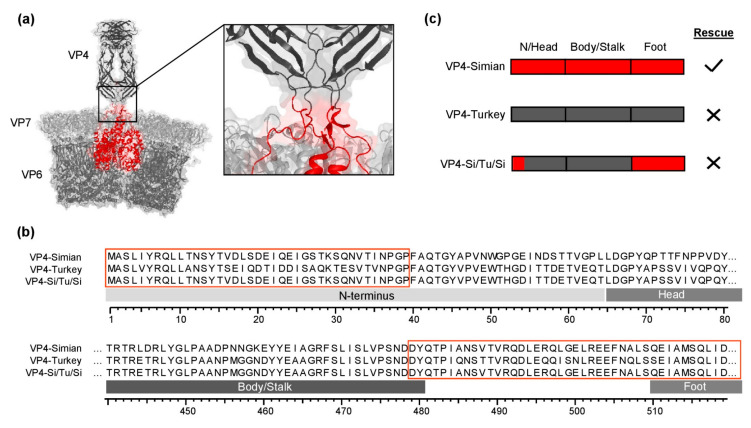
Chimeric simian/turkey VP4. (**a**) Schematic representation of the crystal structure of the rotavirus outer capsid layer based on PDB 4V7Q. The amino acid residues located in cavity formed by VP6 and VP7 are shown in red. (**b**) Amino acid sequence alignment of VP4 from simian SA11 (VP4-Simian) and turkey 2E10 (VP4-Turkey) in comparison to the sequence of the chimeric VP4 construct (VP4-Si-Tu-Si). The red boxes correspond to the amino acid residues shown in red in (**a**). (**c**) Schematic representation of VP4-encoding genome segments used for rescue experiments. N/Head: N-terminus and head region, VP4-Simian: red, VP4-Turkey: dark gray.

Using a plasmid-based reverse genetics system, we have previously shown that VP4 from distantly related RVAs has a high reassortment potential with simian SA11. Among others, viable virus could be generated in this system using VP4 of simian SA11, chicken 2G3, and bat BatLy03. However, we were unable to rescue simian SA11 reassortants with VP4 from turkey 2E10, human Wa, human Moz60a, or human Moz308. While human Moz60a and Moz308 are field strains that have never been cell culture-adapted, turkey 2E10 and human Wa are replicating well in MA-104 cells. The failure to rescue reassortants with VP4 from these strains suggested that unfavorable protein interactions between their VP4 and the VP6/VP7 from SA11 may limit reassortment. To test this hypothesis, we designed a chimeric VP4-encoding genome segment that contained nucleotides from simian SA11 and turkey 2E10. In this construct, the nucleotides encoding amino acid residues that are located in the cavity formed by VP6 and VP7 were derived from simian SA11, while the remaining VP4-encoding nucleotides were from turkey 2E10 (Figure 1a). Figure 1b indicates at which amino acid residues the transition between simian and turkey VP4 occurs. However, we were unable to rescue the reassortant virus using this construct (Figure 1c).

### 3.2. Chimeric Chicken/Turkey VP4

**Figure 2:**
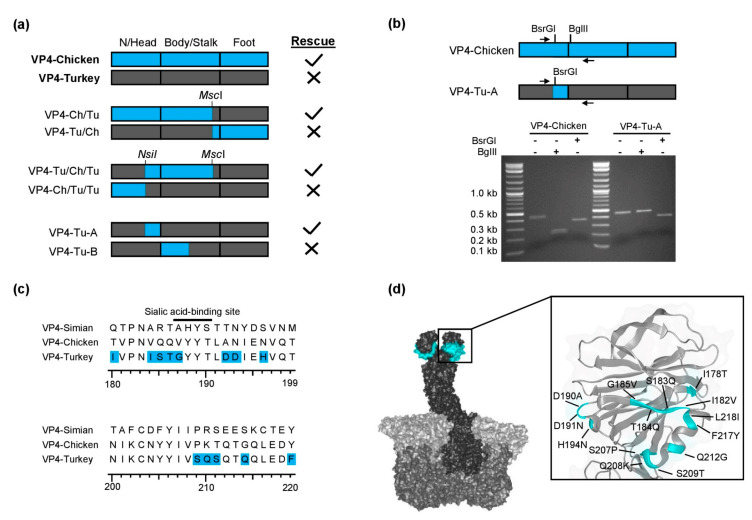
Chimeric chicken/turkey VP4. (**a**) Chimeric VP4-encoding genome segments used for rescue experiments. N/Head: N-terminus and head region, VP4-Chicken: blue, VP4-Turkey: dark gray. (**b**) Agarose gel analysis of a restriction enzyme digestion of RT-PCR products from VP4-Chicken and VP4-Tu-A. The primer-binding sites and restriction enzyme sites are indicated. (**c**) Amino acid sequence alignment of the C-terminal part of the VP4 head region. The amino acid residues in VP4-Tu-A that were substituted with the corresponding residues of VP4-Chicken are highlighted in blue. The black line indicates the position of a known sialic acid-binding site for simian RRV [38]. (**d**) Location of amino acid substitutions (highlighted in cyan) present in the head region of VP4-Tu-A as projected into the 3D structure of simian RRV VP4 (PDB 4V7Q).

In order to identify the regions of VP4 important for the rescue of infectious virus, we constructed chimeric VP4 genes derived from turkey 2E10 and chicken 2G3. These strains share high sequence identities (84.29% on the protein level) and have many restriction enzyme sites on the VP4 gene in common. Both strains replicate in MA-104 cells. However, we were only able to rescue SA11 reassortants with VP4 from chicken 2G3 but not with that from turkey 2E10. To investigate which regions of VP4 limit reassortment, we constructed multiple chimeric VP4-encoding genome segments using available restriction enzyme sites (Figure 2a), which were tested for the generation of replicating virus using the RGS. Surprisingly, the exchange of a central fragment of the VP4-Turkey-encoding genome segment with the corresponding fragment of VP4-Chicken resulted in a replication-competent virus. In this construct (VP4-Tu-Ch-Tu), the nucleotides encoding the C-terminal part of the head region as well as the majority of the body/stalk region were derived from VP4-Chicken (Figure 2a). A sequence alignment showed that the majority of differences between VP4-Turkey and VP4-Chicken were located in the C-terminal end of the head region, the trypsin cleavage site, and the beginning of the body/stalk region.

In order to narrow down which distinct region allowed rescue, we generated two additional chimeric genome segments by splitting the fragment in two parts, VP4-Tu-A and VP4-Tu-B, by cloning synthesized DNA fragments into the vector encoding VP4-Turkey (Figure 2a). Only VP4-Tu-A resulted in replication-competent reassortants, indicating that the head region of the turkey 2E10 VP4 prevented the rescue of replication-competent virus, which could be reverted by using the corresponding region of chicken 2G3 VP4. The presence of the genome segment encoding VP4-Tu-A in the rescued reassortant virus was confirmed by RT-PCR followed by restriction enzyme digestion (Figure 2b). The amino acid residues in VP4-Tu-A that were changed to residues present in VP4-Chicken are highlighted in Figure 2c. Multiple amino acid substitutions were present in the proximity of a known sialic acid-binding site in the head region [38]. The location of the substituted amino acid residues in the 3D structure of VP4 is shown in Figure 2d.

### 3.3. Chimeric Bat/Human VP4

Next, we were interested in exploring the VP4 reassortment restrictions for the three remaining human strains Wa, Moz60a, and Moz308, for which we could not generate reassortants in previous experiments. In order to generate chimeric VP4-encoding genome segments, a bat strain was chosen here, because of compatible restriction enzyme sites, relatively high sequence similarities (>72% identity on the protein level), and reassortants with VP4-Bat were easy to rescue. 

**Figure 3:**
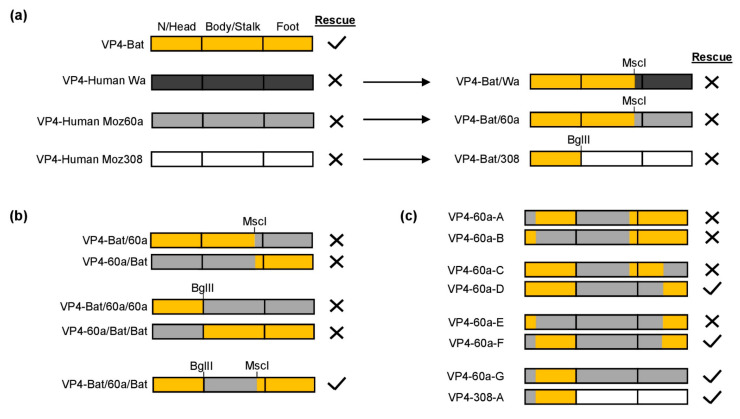
Chimeric bat/human VP4 constructs. N/Head: N-terminus and head region, Bat: orange, human Wa: dark gray, human Moz60a: light gray, human Moz308: white. Successful rescue was confirmed by the presence of a CPE and the detection of viral RNA by qRT-PCR. Negative rescue was confirmed by the absence of a CPE and no detection of viral RNA by qRT-PCR. (**a**) Chimeric genome segments with nucleotides encoding the head region derived from VP4-Bat and the foot region derived from human strains. (**b**) VP4-encoding genome segments with sequences from VP4-Bat and VP4-Human Moz60a (**c**) Genome segments encoding VP4 with different combinations of the N-terminus and foot region.

First, we generated three chimeric plasmids in a similar manner as for turkey/chicken VP4. In VP4-Bat/Wa and VP4-Bat/60a, the N-terminus, head region, and the majority of the body/stalk region were derived from VP4-Bat, while the entire foot region was derived from human Wa or Moz60a, respectively (Figure 3a). In VP4-Bat/308, only the the N-terminus and head region were derived from VP4-Bat, as there were no other comparable restriction enzyme sites available, while the body/stalk and the foot region were from human Moz308 (Figure 3a). Surprisingly, in contrast to the situation in turkey/chicken VP4, none of the chimeric constructs resulted in replication-competent virus.

To further identify responsible regions, we generated additional chimeric genome segments, this time focusing on sequences from VP4-Bat and Moz60a (Figure 3b). The only construct that rescued reassortant virus was VP4-Bat/60a/Bat, in which a fragment encoding the central body/stalk region was derived from VP4-Moz60a, while the remaining nucleotides were derived from the bat strain. This suggested that the N-terminus and the foot region of VP4, which are both located in the cavity formed by VP6 and VP7, could be incompatible in the chimeric protein. 

To investigate this possibility, we designed a series of chimeric constructs that encoded different combinations of the N-terminus and the foot region (Figure 3c). Based on VP4-Bat/60a/Bat, we tested two constructs in which the N-terminus or the head were derived from human Moz60a (VP4-60a-A and VP4-60-B). However, we were unable to rescue either construct. Therefore, we examined the foot region and designed two constructs in which the N-terminal half or the C-terminal half of the foot region were derived from the human strain (VP4-60a-C and VP4-60a-D). Interestingly, VP4-60a-D could be rescued. Based on this modified foot region, the N-terminus was further split (VP4-60a-E and VP4-60a-F). By this, VP4-60a-F resulted in a replication-competent reassortant, indicating that the N-terminus from human VP4-Moz60a was indeed incompatible with the N-terminal sequences of the foot region from VP4-Bat. 

Finally, we could show that the C-terminal part of the foot region in VP4-60a-F could also be substituted with the original human sequences (VP4-60a-G), suggesting that the head region from VP4-Bat alone (without its N-terminus) was sufficient for virus rescue. By placing this fragment (N-terminus of VP4-Moz60a and head region of VP4-Bat) into the residual sequence of VP4-Moz308, we could also rescue viable reassortants (VP4-308-A, Figure 3c). Taken together, this indicates that essentially the same region as in turkey/chicken VP4 is responsible for the restriction of reassortment for the human VP4s.

### 3.4. Chimeric Simian/Human VP4

**Figure 4:**
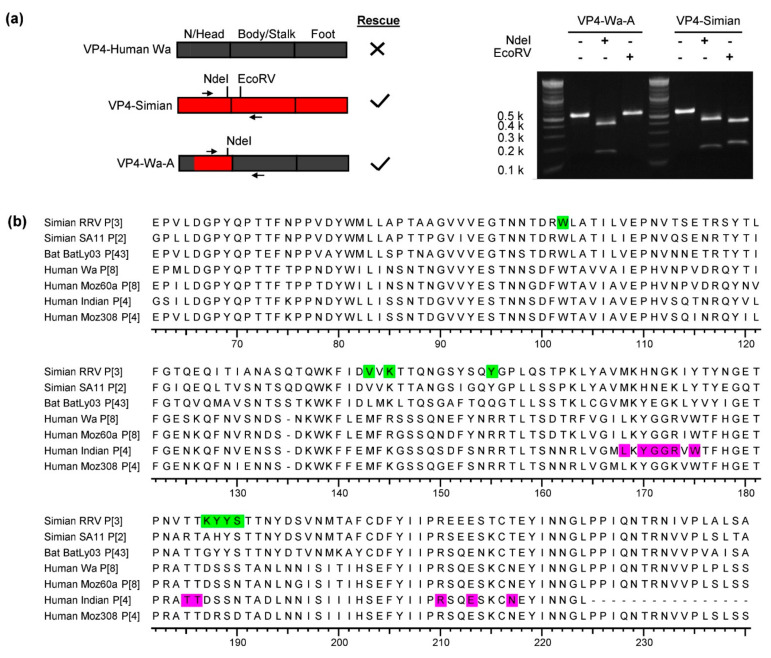
Chimeric simian/human VP4. N/Head: N-terminus and head region, human Wa: dark gray, simian SA11: red. (**a**) Agarose gel analysis of a restriction enzyme digestion of RT-PCR amplicons from VP4-Wa-A and VP4-Simian. The primer-binding sites and restriction sites are indicated left in the schematic overview of the different constructs. (**b**) Amino acid sequence alignment of the substituted head region. The sequences of simian RRV and a human Indian strain are included as their glycan-binding site are known. The known glycan-binding sites of simian RRV VP4 [38] and human Indian VP4 [14] are highlighted in green and purple, respectively.

In order to test if a general mechanism could be responsible for the restriction of VP4 reassortment, the identified fragment of the head region of the human strain Wa was directly exchanged with that of simian SA11 (Figure 4a). By this, a viable reassortant could readily be generated. The presence of the genome segment encoding this chimeric protein, VP4-Wa-A, in the rescued reassortant virus was confirmed by RT-PCR followed by restriction enzyme digestion (Figure 4a). Figure 4b shows a sequence alignment of the amino acid residues encoded by the substituted nucleotides. The residues of VP4 from the three human strains differed considerably in multiple locations in or adjacent to known glycan-binding sites in comparison to VP4-Bat or VP4-Simian.

### 3.5. Growth Kinetics of Cell-Culture Adapted Parent Viruses and their Rescued Reassortant Viruses

**Figure 5:**
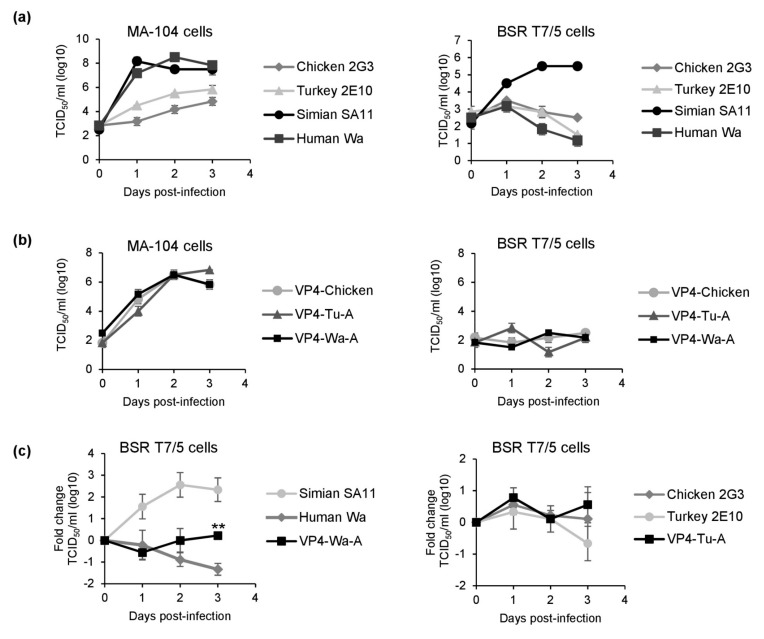
Replication kinetics of the cell culture-adapted parent viruses and their rescued reassortants. MA-104 or BSR-T7/5 cells were infected with 2 × 10^4^ TCID_50_. The TCID_50_ was determined in triplicates at the indicated time points. (**a**) Growth curves of cell-culture adapted viruses. Data are means plus/minus SD (**b**) Growth curves of reassortants. Data are means plus/minus SD. (**c**) Comparison of titer changes of parent viruses and reassortants in BSR-T7/5 cells. The TCID_50_ was normalized to the value from day 0 and the fold change was calculated. TCID_50_ values at day 0 were: 1.94 log_10_ for simian SA11, 2.83 log_10_ for human Wa, 2.17 log_10_ for VP4-Wa-A, 2.28 log_10_ for turkey 2E10, 1.94 log_10_ for chicken 2G3, and 1.72 log_10_ for VP4-Tu-A. Data are means plus/minus SD from three independent experiments. ** *p* < 0.01 for human Wa vs. VP4-Wa-A.

The head region of VP4 contains a receptor-binding site. While the structural compatibility of the N-terminus and the foot region seemed to be relevant, substituting the nucleotides in the head region alone allowed us to rescue the four VP4 reassortants that we were previously not able to rescue. This included VP4 from two strains adapted to efficient growth in MA-104 cells, suggesting that growth in this cell line is probably not the main restriction factor. However, the RGS also uses BSR T7/5 cells, which might show a different repertoire of surface molecules. To investigate this possibility, we examined the replication kinetics of the cell culture-adapted viruses (simian SA11, human Wa, chicken 2G3, and turkey 2E10) in MA-104 cells and in BSR T7/5 cells (Figure 5a). The cells were infected with the same infectious doses, and the TCID_50_ was determined from culture supernatants taken at day 0, 1, 2, and 3 post infection. The experiment showed that all viruses replicated in MA-104 cells. The two avian rotaviruses, chicken 2G3 and turkey 2E10, replicated to lower titers than simian SA11 and human Wa. In contrast, clear replication in BSR T7/5 cells was only observed for simian SA11. The infectious titer of chicken 2G3 remained relatively stable indicating low-level replication, while the titers for turkey 2E10 and human Wa decreased after day 1, which was indicative of no replication (Figure 5a). 

Next, we examined the replication kinetics of the new reassortants VP4-Tu-A and VP4-Wa-A and a previously characterized reassortant, VP4-Chicken (Figure 5b). All reassortants replicated in MA-104 cells. However, similar to what was observed for chicken 2G3, the infectious titers of all reassortants remained relatively stable in BSR T7/5 cells over the time course of the experiment, indicating low-level replication. To further investigate the replication kinetics, we directly compared the titer changes of the parent viruses and the new reassortants in BSR T7/5 cells (Figure 5c), confirming that the titer of human Wa continuously decreased, while the titer of the chimeric reassortant VP4-Wa-A remained relatively stable (*p* < 0.01). We observed a similar trend for the avian viruses and new reassortant. The titer of chicken 2G3 and VP4-Tu-A remained stable, while the titer for turkey 2E10 was reduced at the end of the experiment. However, this effect did not reach statistical significance.

## 4. Discussion

Using a plasmid-based RGS, we have previously been unable to rescue reassortants with VP4 from the cell culture-adapted turkey strain 2E10, the cell culture-adapted human strain Wa, and two unadapted human field strains (Moz 60a and Moz308) in the backbone of simian SA11. Here, we have shown that substituting the nucleotides encoding the head region of VP4 enabled the rescue of replication-competent reassortants for all four strains. In contrast, the body/stalk region as well as the N-terminus and the foot region, which interact with VP6 or VP7, are not strictly limiting the VP4 reassortment. However, structural compatibility of the N-terminus and the foot region also seemed to be important for rescuing the reassortants. Analysis of the replication kinetics of the cell culture-adapted parent viruses and rescued reassortants in BSR T7/5 cells revealed that only simian SA11 replicated to higher titers, while no replication was evident for the cell culture-adapted turkey strain 2E10 and the human strain Wa. However, we observed minimal replication of the chimeric rescued reassortants, which could indicate that their slow replication in the transfected cell line aids their rescue by the RGS. Additionally, the chimeric VP4 proteins could have enhanced attachment to MA-104 cells. Future research is necessary to support either hypothesis.

The generation of mono-reassortants with human VP4 or human VP7 in a simian SA11 backbone has been recently described [28,29,30,36]. While VP7 was readily interchangeable, the rescue of VP4 reassortants was more difficult. We rescued one simian SA11-based reassortant with VP4 from a field strain that has never been adapted to cell culture [36]. In comparison to the simian parent virus, this VP4 reassortant replicated significantly slower. Kawagishi et al. also observed that a simian SA11-based reassortant with VP4 from a cell culture-adapted human RVA replicated, but to lower titers than either the simian or the human parent virus [30]. Kanai et al. have very recently shown that substituting the entire receptor-binding VP8* fragment, which contains the N-terminus and the head region, improved the replication of a chimeric reassortant with human/simian VP4 [29]. Our results confirm that amino acid residues in the head region are important for rescuing replication-competent reassortants. However, we show for VP4 from four different RVAs that it is not necessary to substitute the nucleotides encoding the N-terminal amino acid residues. In some of our chimeric constructs, substituting these nucleotides even prevented the rescue of replication-competent reassortants, indicating that strain-specific amino acid residue differences in the N-terminus can affect the interaction of the N-terminus with the foot region. These results suggest that, in addition to amino acid residues in the head region, the structural compatibility of the N-terminus and the foot region is also relevant for the rescue of chimeric VP4. 

The two major rotavirus vaccines Rotarix (GlaxoSmithKline Biologicals, Belgium) and RotaTeq (Merck & Co., Inc., United States) cover only a limited number of P-types. Rotarix is based on an attenuated human G1P[8] strain, while Rotateq contains one reassortant carrying human VP4 from a P1A[8] strain [39,40,41]. The VP5* fragment contains multiple neutralizing epitopes, and anti-VP5* antibodies can mediate protection from RVA-associated diarrhea in suckling mice [42,43]. While VP5* is relatively well conserved, different neutralizing epitopes in human VP5* have been identified [44]. Komoto et al. have previously generated a human KU-based reassortant with simian VP4, in which one cross-reactive neutralization epitope in the VP5* fragment was derived from human DS-1 [45]. Here, we have generated three rescued reassortants that contain VP4 with the entire human VP5* fragment. This approach could be useful to generate vaccine strains with a diverse set of human VP5* fragments that differ in their neutralizing epitopes. However, additional experiments are needed to confirm the usefulness of this approach for the development of novel vaccine strains. 

It is unknown whether VP4 from the primary human isolates that have never been adapted to cell culture can mediate efficient infection of MA-104 cells. Therefore, substituting the head region may have resulted in better attachment to MA-104 cells. In contrast, VP4 from the cell culture-adapted strains turkey 2E10 and human Wa can clearly mediate entry in MA-104 cells. Therefore, the result that substituting the head region was necessary to enable rescue of reassortants with VP4 from these strains was, to a certain degree, unexpected. The ability to rescue rotaviruses using the plasmid-based RGS seems to depend on how many infectious virus particles are produced following transfection and how well the produced particles can infect the receiver cell line that is used to amplify the produced viruses. Komoto et al. have rescued human strain KU using reverse genetics [26]. Rescue was only possible after the roller-tube culture technique, with MA-104 cells was applied to amplify the recombinant human RVA. Kawagishi et al. used additional helper plasmids to increase virus production in BSR T7/5 cells for rescuing human strain Odelia [30], while Sanchez-Tacuba et al. (2020) used a different helper plasmid to increase virus production after transfection and a modified MA-104 cell line that is more susceptible to rotavirus infection for rescuing human strain CDC-9 and other difficult to rescue strains [31]. For rescuing RRV, using a different helper plasmid seemed to have a much better effect than using a more susceptible cell line [31]. We have shown that in contrast to simian SA11, human Wa is unable to replicate in BSR T7/5 cells. The ability to replicate in BSR T7/5 cells could be one factor that influences the number of infectious particles prior to infection of the receiver cell line. Therefore, future studies could focus on an improvement of the existing T7-RNA-polymerase expressing cell lines or on the use of alternative cell lines that are permissive for the replication of human RVAs in order to increase the rescue efficiency. As an example, HEK293 cells could be a viable option as they are highly transfectable, express large amounts of proteins, and are commonly used to produce other viruses. At least for one human strain, WI61 (G9P[8]), infection of HEK293 was shown [17]. Culture conditions may also affect replication of RVAs in cultured cells. For example, the addition of calcium to the culture medium was shown to increase replication for a panel of different RVA strains [46]. Therefore, optimizing media compositions in the cell line used for transfection or the receiver cell line used for amplification could be another point for optimization. 

Additionally, we observed higher titers for the rescued reassortant with chimeric human/simian VP4 in comparison to human Wa in BSR-T7/5 cells. This minor difference could have been sufficient to allow productive infection of the receiver cell line. A comparison of the growth kinetics of the other reassortants with the bat strain BatLy03 and the primary human strains in BSR T7/5 cells would have strengthened this point. However, these strains have never been isolated in cell culture, and we have no access to the source material. Additional experiments, such as virion binding experiments to different target cells, are needed to support the hypothesis that substituting the head region allowed replication in BSR T7/5 cells. Another possible explanation for being able to rescue the reassortants could be that chimeric VP4 improved attachment to MA-104 cells. While we have shown that the viral protein–protein interactions did not strictly limit the generation of VP4 reassortants, we cannot exclude that they still influence the efficiency of VP4 reassortant rescue. Pesavento et al. observed subtle alterations in the conformation of VP4 in certain VP4 reassortants [47], which could negatively affect the ability to bind to the natural receptor used in MA-104 cells. Additionally, analyses of circulating human RVAs suggest that there are preferred genome constellations [48]. Interaction of the viral untranslated regions at the genome segment ends seem unlikely to be the cause for preferred genome constellations as they are highly conserved among RVAs [49,50]. On the other hand, Heiman et al. have shown that human RVA genome constellations are influenced by viral protein interactions and identified genotype specific changes in VP2–VP6, VP4–VP6, and VP7–VP6 interfaces [51]. Therefore, further studies should analyze those effects in reassortants and rule out their effect on virus rescue and replication efficiency. 

Overall, we have shown that a region in VP4 containing the receptor-binding site was restricting the rescue of four reassortants that we were previously unable to rescue. Substituting nucleotides encoding this region with the corresponding nucleotides from strains that we were previously able to rescue enabled us to generate replication-competent reassortants. This may be linked to the ability of VP4 to mediate the infection of one of the cell lines used for the generation of the reassortants in the applied RGS or improved binding to the cell line that is used to amplify the recombinant virus. Additional experiments are necessary to support either hypothesis. Knowledge about genome regions critical for the rescue of efficiently replicating reassortants may be useful for basic and applied science, including the targeted generation of defined rotaviruses for diagnostic applications and vaccines.

## Data Availability

Not applicable.

## Supplementary Materials

The following are available online at https://www.mdpi.com/1999-4915/13/3/363/s1, Table S1: Unmodified VP4-encoding genome segments used in this study, Table S2: Chimeric VP4 genome segments generated in this study. Table S3: Amino acid residues in chimeric VP4.
