# Supplementary material for: Rescue of Infectious Rotavirus Reassortants by a Reverse Genetics System Is Restricted by the Receptor-Binding Region of VP4
Source: Viruses. 2021 Feb 25;13(3):363. doi: 10.3390/v13030363 (PMC7996497; doi:10.3390/v13030363)
Supplement: Supplementary file 1 [file viruses-13-00363-s001.pdf]

Supplemental Table S1. Unmodified VP4-encoding genome segments used in this study.

Strain	Genbank number	Designation of VP4 segment
RVA/Simian-tc/ZAF/SA11-L2/1958/G3P[2]	LC333805.1	VP4-Simian
RVA/Bat-wt/CMR/BatLy03/2014/G25P[43]	KX268779.1	VP4-Bat
RVA/Human-wt/MOZ/0060a/2012/G12P[8]	MG926761.1	VP4-Human Moz60a
RVA/Human-wt/USA/Wa/1974/G1P[8]	KT694942.1	VP4-Human Wa
RVA/Human-wt/MOZ/ 0308/2012/G2P[4]	MG926728.1	VP4-Human Moz308
RVA/Turkey-tc/GER/03V0002E10/2003/G22P[35]	JX204825.1	VP4-Turkey
RVA/Chicken-tc/GER/02V0002G3/2002/G19P[30]	KT239165.1	VP4-Chicken

Supplemental Table S2. Chimeric VP4 genome segments generated in this study. The nucleotide numbers indicate the position of the substituted fragments in the original unmodified genome segment. It is of note that not all VP4-encoding genome segments have the same size, but no additional nucleotides were introduced or deleted because of the cloning strategy. VP4-60a and VP4-308 refer to the VP4 segment of human strains Moz60a and Moz308, respectively.

Chimeric segment	Nucleotides
VP4-Si/Tu/Si	1-144 VP4-Simian/145-1437 VP4-Turkey/1450-2362 VP4-Simian
VP4-Tu/Ch	1-1179 VP4-Turkey/1180-2353 VP4-Chicken/
VP4-Ch/Tu	1-1179 VP4-Chicken/1180-2353 VP4-Turkey
VP4-Tu/Ch/Tu	1-501 VP4-Turkey/502-1179 VP4-Chicken/1180-2353 VP4-Turkey
VP4-Ch/Tu/Tu	1-501 VP4-Chicken/502-2353 VP4-Turkey
VP4-Bat/Wa	1-1191 VP4-Bat/1189-2359 VP4-Wa
VP4-Bat/60a	1-1191 VP4-Bat/1189-2359 VP4-60a
VP4-Bat/308	1-1191 VP4-Bat/1189-2359 VP4-308
VP4-60a/Bat	1-1188 VP4-60a/1192-2362 VP4-Bat
VP4-60a/Bat/Bat	1-727 VP4-60a/731-2362 VP4-Bat
VP4-Bat/60a/60a	1-730 VP4-Bat/728-2359 VP4-60a
VP4-Bat/60a/Bat	1-730 VP4-Bat/728-1188 VP4-60a/1192-2362 VP4-Bat
VP4-Tu-A	1-501 VP4-Turkey/502-675 VP4-Chicken/676-2353 VP4-Turkey
VP4-Tu-B	1-675 VP4-Turkey/676-924 VP4-Chicken/925-2353 VP4-Turkey
VP4-60a-A	1-192 VP4-60a/193-730 VP4-Bat/728-1188 VP4-60a/1192-2362 VP4-Bat
VP4-60a-B	1-201 VP4-Bat/202-1188 VP4-60a/1192-2362 VP4-Bat
VP4-60a-C	1-730 VP4-Bat/728-1188 VP4-60a/1192-1917 VP4-Bat/1915-2359 VP4-60a
VP4-60a-D	1-730 VP4-Bat/728-1905 VP4-60a/1909-2362 VP4-Bat
VP4-60a-E	1-201 VP4-Bat/202-1905 VP4-60a/1909-2362 VP4-Bat
VP4-60a-F	1-192 VP4-60a/193-730 VP4-Bat/728-1905 VP4-60a/1909-2362 VP4-Bat
VP4-60a-G	1-192 VP4-60a/193-730 VP4-Bat/728-2359 VP4-60a
VP4-308-A	1-192 VP4-60a/193-730 VP4-Bat/728-2359 VP4-308
VP4-Wa-A	1-192 VP4-Wa/193-730 VP4-Simian/728-2359 VP4-Wa

Supplemental Table S3. Amino acid residues in chimeric VP4. The amino acid residue numbers indicate which residues were derived from the indicated strains in the final chimeric VP4 protein. It is of note that not all VP4s have the same size, but no additional amino acid residues were introduced or deleted because of the cloning strategy. VP4-60a and VP4-308 refer to the VP4 segment of human strains Moz60a and Moz308, respectively.

Chimeric segment	Amino acid residues
VP4-Si/Tu/Si	1-45 VP4-Simian/46-476 VP4-Turkey/477-773 VP4-Simian
VP4-Tu/Ch	1-390 VP4-Turkey/391-771 VP4-Chicken/
VP4-Ch/Tu	1-390 VP4-Chicken/391-771 VP4-Turkey
VP4-Tu/Ch/Tu	1-164 VP4-Turkey/165-390 VP4-Chicken/391-771 VP4-Turkey
VP4-Ch/Tu/Tu	1-164 VP4-Chicken/165-771 VP4-Turkey
VP4-Bat/Wa	1-393 VP4-Bat/394-777 VP4-Wa
VP4-Bat/60a	1-393 VP4-Bat/394-777 VP4-60a
VP4-Bat/308	1-240 VP4-Bat/241-777 VP4-308
VP4-60a/Bat	1-393 VP4-60a/394-776 VP4-Bat
VP4-60a/Bat/Bat	1-239 VP4-60a/240-776 VP4-Bat
VP4-Bat/60a/60a	1-240 VP4-Bat/241-777 VP4-60a
VP4-Bat/60a/Bat	1-240 VP4-Bat/241-394 VP4-60a/395-777 VP4-Bat
VP4-Tu-A	1-164 VP4-Turkey/165-222 VP4-Chicken/223-771 VP4-Turkey
VP4-Tu-B	1-222 VP4-Turkey/223-305 VP4-Chicken/306-771 VP4-Turkey
VP4-60a-A	1-61VP4-60a/62-240 VP4-Bat/241-394 VP4-60a/395-777 VP4-Bat
VP4-60a-B	1-64 VP4-Bat/65-393 VP4-60a/394-776 VP4-Bat
VP4-60a-C	1-240 VP4-Bat/241-394 VP4-60a/395-636 VP4-Bat/637-777 VP4-60a
VP4-60a-D	1-240 VP4-Bat/241-633 VP4-60a/634-777 VP4-Bat
VP4-60a-E	1-64 VP4-Bat/65-632 VP4-60a/633-776 VP4-Bat
VP4-60a-F	1-61 VP4-60a/62-240 VP4-Bat/241-633 VP4-60a/634-777 VP4-Bat
VP4-60a-G	1-61 VP4-60a/62-240 VP4-Bat/241-777 VP4-60a
VP4-308-A	1-61 VP4-60a/62-240 VP4-Bat/241-777 VP4-308
VP4-Wa-A	1-61 VP4-Wa/62-240 VP4-Simian/241-777 VP4-Wa
